# Secondary Distribution of Dual HIV/Syphilis Self-Testing Among Men Who Have Sex With Men: Pragmatic Randomized Controlled Trial in China

**DOI:** 10.2196/70775

**Published:** 2025-09-25

**Authors:** Min Wang, Yun Zhang, Weiyi Tian, Jinli Mo, Haimei Huang, Jiawen Zhu, Sumin Tan, Yingqiong Huang, Li Jiang, Ping Cen, Guanghua Lan, Hao Wang, Wei Pan, Joseph D Tucker, Chuanyi Ning

**Affiliations:** 1 Nursing College Guangxi Medical University Nanning China; 2 The Second Affiliated Hospital of Guangxi Medical University Nanning China; 3 Department of Infection Disease The Fourth People's Hospital of Nanning Nanning China; 4 Department of AIDS/STD Control and Prevention Nanning Municipal Center for Disease Prevention and Control Nanning China; 5 Institute of HIV/AIDS Prevention and Control Guangxi Zhuang Autonomous Region Center for Disease Control and Prevention Nanning China; 6 Institute of Biomedicine Department of Infectious Diseases University of Gothenburg Gothenburg Sweden; 7 Duke University School of Nursing Durham, NC United States; 8 Department of Population Health Sciences Duke University School of Medicine Durham, NC United States; 9 Department of Medicine University of North Carolina at Chapel Hill Chapel Hill, NC United States; 10 Faculty of Infectious and Tropical Diseases London School of Hygiene and Tropical Medicine London United Kingdom; 11 Guangxi Key Laboratory of AIDS Prevention and Treatment Nanning China

**Keywords:** HIV, syphilis, men who have sex with men, self-testing

## Abstract

**Background:**

The World Health Organization recommends dual HIV/syphilis testing, but this approach has not been examined in many low- and middle-income countries. Dual HIV/syphilis self-testing may accelerate secondary distribution of self-test kits. Preliminary studies in Guangdong, China, have demonstrated the feasibility and cost-effectiveness of dual HIV/syphilis self-testing distribution via social media, but evidence comparing dual HIV/syphilis self-testing to single HIV self-testing for secondary distribution within social networks remains limited in resource-limited settings.

**Objective:**

We aimed to evaluate the effectiveness of secondary distribution of dual HIV/syphilis self-testing kits in promoting HIV testing uptake among men who have sex with men (MSM) in China.

**Methods:**

We conducted a pragmatic 3-arm randomized controlled study in the Guangxi Zhuang Autonomous Region, China. MSM aged 18 years or older who were HIV-negative were enrolled and randomly assigned (1:1:1) to either the site-based HIV testing (SBT) group (110/330, 33.3%), single HIV self-testing (SST) group (110/330, 33.3%), or dual HIV/syphilis self-testing (DST) group (110/330, 33.3%). Participants in the SST and DST groups received free finger-prick-based HIV self-testing or HIV/syphilis self-testing kits at enrollment and during the 12-month follow-up. The primary outcome was the mean number of social network members motivated by the participant and the mean frequency of HIV tests per participant within a 3-month period. The data were analyzed using an intention-to-treat analysis.

**Results:**

A total of 330 MSM were recruited, among whom 319 (319/330, 96.7%) completed at least 1 follow-up survey and were subsequently included in the analysis. Among the participants, 245/319 (77%) had a college education or above. Compared to social network members in the SBT group, those in the intervention SST and DST groups were more likely to motivate others for HIV testing over a 3-month average duration. The mean number of motivated individuals was 0.42 in the SST group versus 0.20 in the SBT group, a mean difference (MD) of 0.22 (95% CI 0.12-0.33; *P*<.001). The mean was 0.51 in the DST group versus 0.20 in the SBT group, with an MD of 0.32 (95% CI 0.20-0.43; *P*<.001). The mean frequency of total HIV tests per participant in the SST group (1.33) was higher than that in the SBT group (0.87), with an MD of 0.46 (95% CI 0.31-0.62; *P*<.001) over 3 months. Over a 3-month period, the mean number of HIV tests per participant was higher in the DST group (1.43) than in the SBT group (0.87), with an MD of 0.57 (95% CI 0.41-0.73; *P*<.001). A total of 4 (1.3%) individuals had a new HIV positive result, while 11 (3.4%) had a new syphilis positive result. All individuals who had positive self-test results underwent laboratory-based confirmation tests. There were no adverse events reported.

**Conclusions:**

Our data demonstrate that the secondary distribution strategy of HIV/syphilis self-testing proves to be an effective means of expanding HIV testing coverage by encouraging the distribution of testing kits within the social networks of MSM.

**Trial Registration:**

Chinese Clinical Trial Registry ChiCTR2100050898; https://www.chictr.org.cn/hvshowproject.html?id=158876

## Introduction

The syndemic relationship between HIV and syphilis among men who have sex with men (MSM) is well-documented, with each infection potentiating the transmission and progression of the other [1,2]. Syphilis increases the risk of HIV acquisition by 2- to 5-fold through mucosal inflammation and immune activation, while HIV accelerates syphilis progression and complicates clinical management [3,4]. The World Health Organization (WHO) recommends dual HIV/syphilis rapid diagnostic tests to screen high-risk populations, thereby reducing undiagnosed infection, untreated morbidity, and mortality [5]. This integrated approach is particularly vital in low- and middle-income countries (LMICs) like China, where key populations face disproportionate burdens. Recently, a systematic review and meta-analysis showed that the prevalence of HIV/syphilis coinfection among MSM in China was 2.7% [6].

Despite WHO guidance, dual testing uptake remains alarmingly low. A cross-sectional study revealed that only 37% (95% CI 34%-40%) of Chinese MSM who had ever tested for HIV received dual screening [7]. Several barriers impede MSM from facility-based HIV testing at hospitals or Centers for Disease Control and Prevention (CDC) clinics, including HIV-related stigma, inconvenient testing times at clinics, and prolonged testing waiting times, all of which decrease HIV test uptake [8,9]. According to the Joint United Nations Programme on HIV/AIDS (UNAIDS) data, 13% of people living with HIV in the world were still unaware of their infection status in 2024 [10]. The population of undiagnosed people living with HIV in China is still large, at an estimated 15.7% in 2023 [11].

HIV self-testing (HIVST) has emerged as a transformative strategy to overcome these barriers. By enhancing privacy and convenience and decreasing stigma, HIVST enables people to test autonomously and expands coverage among hard-to-reach populations [12,13]. Building on this innovation, dual HIV/syphilis self-testing represents a significant advancement. Evidence confirms that dual screening for HIV and syphilis is acceptable to MSM and more cost-effective than separate rapid tests for each infection [14,15]. A European clinic-utility study evaluated dual point-of-care tests for HIV/syphilis across community-based organizations in Spain, Slovenia, Latvia, and Ukraine and reported high acceptability and usability among both MSM users and providers [16]. Considering the known synergy between HIV and syphilis, dual HIV/syphilis self-testing could be useful for clinical and public health reasons.

Research that explores the effect of dual HIV/syphilis self-testing secondary distribution among MSM in China is limited. Social network–based HIV testing approaches involve individuals motivating their sexual or social contacts to take HIV tests [17]. HIVST secondary distribution is a method rooted in social networks, where individuals receive multiple HIVST kits and distribute them to people in their social networks, including sexual partners and friends [18]. The distribution of HIVST kits within social networks can expand HIV testing coverage among MSM. A secondary data analysis reported the distribution of HIVST kits to 647 individuals, 54.6% of whom were first-time testers [19]. In a cohort study, HIVST kits were distributed to 319 Nigerian MSM, 17.9% of them being first-time testers [20]. Given the synergy between HIV and syphilis, dual self-testing could optimize the public health impact. Recent studies in Guangdong, China, highlight the promise of dual HIV/syphilis self-testing for MSM. Wu et al [21] showed that social media-based dual HIV/syphilis self-testing distribution increased testing uptake and case identification, while Wang et al [22] confirmed its cost-effectiveness in a multiarm randomized controlled trial (RCT). However, none have compared dual HIV/syphilis self-testing against single HIVST within a social network or assessed scalability in resource-limited settings. This gap is significant, given the synergy between HIV and syphilis and the potential for secondary distribution to reach hidden high-risk subgroups. To address this gap, we conducted the first pragmatic 3-arm RCT to examine whether the secondary distribution of dual HIV/syphilis self-testing kits promotes HIV testing uptake among Chinese MSM, compared to single HIVST and site-based HIV testing.

## Methods

### Study Design and Participants

The full study protocol has been previously published [23]. This study was designed as a parallel, open-label, 3-arm RCT and adhered to the CONSORT (Consolidated Standards of Reporting Trials) guidelines (Multimedia Appendix 1). The trial was conducted in collaboration with 4 community-based organizations (CBOs) serving MSM populations across 4 cities in Guangxi, China (Nanning, Guilin, Liuzhou, and Beihai). Each CBO operated within government-supported HIV testing clinics at local CDC facilities and provided MSM-friendly health services, including HIV/STI testing, counselling, and linkage to care. Additionally, the CBOs utilize online platforms (WeChat public accounts and Blued, a gay-friendly social network app) for health promotion. Participants were recruited through convenience sampling between September 1, 2021, and February 28, 2022. The follow-up was completed by February 29, 2023. Our multichannel recruitment strategy included online recruitment through study advertisements (with QR codes) on CBOs’ WeChat public accounts and Blued, as well as on-site recruitment at CBO offices, CDC clinics, and MSM-frequented venues (eg, bars and parks) where trained staff approached potential participants.

MSM were eligible if they (1) were biologically male, (2) aged ≥18 years, (3) reported engaging in anal sex with male sex partners, (4) tested negative for HIV and syphilis by rapid screening testing, (5) resided in Guangxi, and (6) were willing to provide personal information and informed consent. Participants were excluded if they met either of the following criteria: (1) presented with evident mental illness or intellectual disability, or (2) encountered language and communication barriers hindering their ability to effectively participate and complete the study.

### Randomization and Masking

Participants deemed eligible were assigned, in a 1:1:1 allocation ratio, to one of the 3 groups: the site-based HIV testing (SBT or control) group, the single HIV self-testing (SST) group, and the dual HIV/syphilis self-testing (DST) group. This allocation was achieved through a simple randomization process using the computer-generated tables in SPSS software (version 29.0; IBM Corp). An external research assistant, uninvolved in the trial, securely sealed the study allocations within sequentially numbered opaque envelopes. Recruitment staff and participants were not informed about study arm allocation until the opening of the sealed envelope following informed consent and enrollment. The statisticians responsible for the data analysis were blinded to participant assignment throughout the study.

### Procedures

Potential participants completed a 3-step screening process. First, prescreening was conducted online or face-to-face to verify inclusion criteria (biological male, age ≥18 years, reported anal sex with male sex partners, and Guangxi residency). Second, eligible individuals underwent onsite rapid HIV/syphilis testing at CBOs, with only double-negative participants proceeding. Third, these individuals provided written informed consent and completed the baseline survey during the baseline assessment phase.

Upon enrollment, participants assigned to the SBT group underwent HIV testing at local hospitals and free HIV testing and counselling clinics at local CDCs and CBOs. Additionally, they had the option to purchase single HIVST kits or dual HIV/syphilis self-testing kits from nearby pharmacies or through online platforms. For the SBT group, personalized online virtual cards featuring a unique identification number and QR code were provided every 3 months via WeChat. Participants could upload their HIV test results by scanning the QR code embedded in the virtual card. Participants were encouraged to distribute the online cards to their social network members (friends and sexual partners) to undergo HIV testing at local hospitals, CDCs, and CBOs.

Participants in the SST group or DST group paid a refundable deposit (US $4) to receive 2 single HIVST kits or dual HIV/syphilis self-testing kits along with a self-testing instructional video via WeChat. When participants uploaded their self-testing results through WeChat, contacted a research assistant, or scanned the QR code in the kits, the deposit was refunded. Participants could order additional self-testing kits after returning the results from their self-test kits during the 12-month study follow-up period. The self-testing kits were capped at 2 kits each. Participants were also encouraged to distribute self-testing kits to their social network members, including friends and sexual partners. To protect participants’ privacy and accommodate their preferences, the test kits were dispatched via a confidential package through postal services, without any reference to HIV.

We used the finger-prick Determine HIV 1/2 early detect test kit (Alere Medical Co) and the SD Bioline HIV/syphilis Duo rapid test kit (Abbott Rapid Diagnostics) in this study. These 2 kits have high specificity and sensitivity. For the Determine HIV 1/2 test, the specificity and sensitivity are 99.75% and 100%, respectively [24]. For the Bioline HIV/syphilis duo, the specificity and sensitivity for HIV-1/2 are 99.67% and 99.91%, and for syphilis, 99.72% and 99.67%, respectively [25]. The syphilis component of the Bioline HIV/syphilis Duo test detects antibodies against *Treponema pallidum* (IgG and IgM), which indicates exposure to syphilis but cannot differentiate between current and past infections. The interpretation of the test results requires a minimum waiting period of 15 minutes, which can extend to 60 minutes. All participants received structured pretest counselling delivered face-to-face or online by trained CBO staff. The pretest counselling covered information on HIV and syphilis transmission and prevention, step-by-step instructions on how to perform the self-test, interpretation of possible results (including the possibility of false positives/negatives), risk reduction strategies, and emergency contact information. For participants and their social network members who got a positive HIV or syphilis self-test result, immediate posttest telephone counselling was provided by trained CBO staff within 2 hours of the result upload. The posttest counselling included emotional support, interpretation of the reactive results, instructions for laboratory confirmation at designated CDC clinics/hospitals within 48 hours, and prebooked appointments. All confirmed positive cases were linked to treatment through the national HIV/syphilis control program.

Data were collected through face-to-face or online questionnaire interviews that were approximately 15 minutes in length. The baseline questionnaire covered sociodemographic information, past HIV/syphilis testing, HIVST, and the frequency of HIV testing, including both HIVST and site-based HIV testing, among MSM in the past 12 months. Follow-up questionnaires at 3, 6, 9, and 12 months covered the frequency and results of HIV testing, distribution of self-testing kits, and sexual behaviors.

Participants received a maximum subsidy of US $27 for completing study surveys: baseline surveys, follow-up surveys at 3 and 6 months (US $15), and follow-up surveys at 9 and 12 months (US $12).

### Outcomes

The primary outcome was the average number of social network members that each participant motivated to test for HIV and the mean frequency of total HIV testing within a 3-month period (HIV testing included both facility-based HIV testing and self-testing). Frequencies were determined by uploading photos of HIV test results and self-reporting test frequency, excluding instances of self-testing with invalid results. The former was determined by uploading photos of HIV test results and self-reporting. The secondary outcomes were HIV and syphilis positive results.

### Ethical Considerations

This study was reviewed and approved by the Medical Ethics Committee of Guangxi Medical University, China (20210173). Written informed consent was obtained from all participants before interviews and study procedures. Privacy and confidentiality were protected through deidentification of study data, secure double-data entry using EpiData software (version 3.1; EpiData Association), and dispatch of test kits via confidential postal packages without HIV-related identifiers. Participants received compensation totaling up to US $27 for survey completion (US $15 for baseline, 3-month, and 6-month surveys, and US $12 for 9-month and 12-month surveys). No images of the participants are included in the manuscript or supplementary materials.

### Statistical Analysis

The required study sample size was determined using the PASS software (version 15.0; NCSS) based on the HIV testing frequency among MSM. It was assumed that the intervention groups would exhibit a higher frequency of HIV testing compared to the control group. Based on the literature [26], the control group had a mean of 1.40 (SD 1.13) and the intervention group had a mean of 2.18 (SD 1.77), which were adopted as the anticipated effect sizes for this study. With an alpha level of 0.05, a statistical power of 0.90, and assuming a conservative 10% loss to follow-up, it was determined that a minimum of 99 participants per arm, totaling 330 MSM, would provide adequate statistical power.

Data were double-entered into Epidata software. The primary and secondary outcomes were analyzed using intention-to-treat analysis, and participants were included in the complete case analysis if they initiated at least 1 follow-up survey. Baseline characteristics were reported using descriptive statistics. Categorical variables were presented as counts and relative proportions. Continuous variables were expressed as the median and IQR. The mean number of social network members motivated by participant and HIV test frequency across generalized linear mixed models was compared, and effect sizes were estimated using mean difference (MD) and risk difference (RD) for primary and secondary outcomes. A 95% CI for MD and RD was calculated. All statistical analyses were conducted using SPSS software (version 26.0; IBM Corp). For multiple comparisons involving the 3 study arms, Bonferroni correction was applied by dividing the alpha level (0.05) by the number of outcome comparisons (n=3), resulting in a significance threshold of *P*<.017. The study was registered with the Chinese Clinical Trial Registry (ChiCTR2100050898).

## Results

### Participant Characteristics

**Figure 1:**
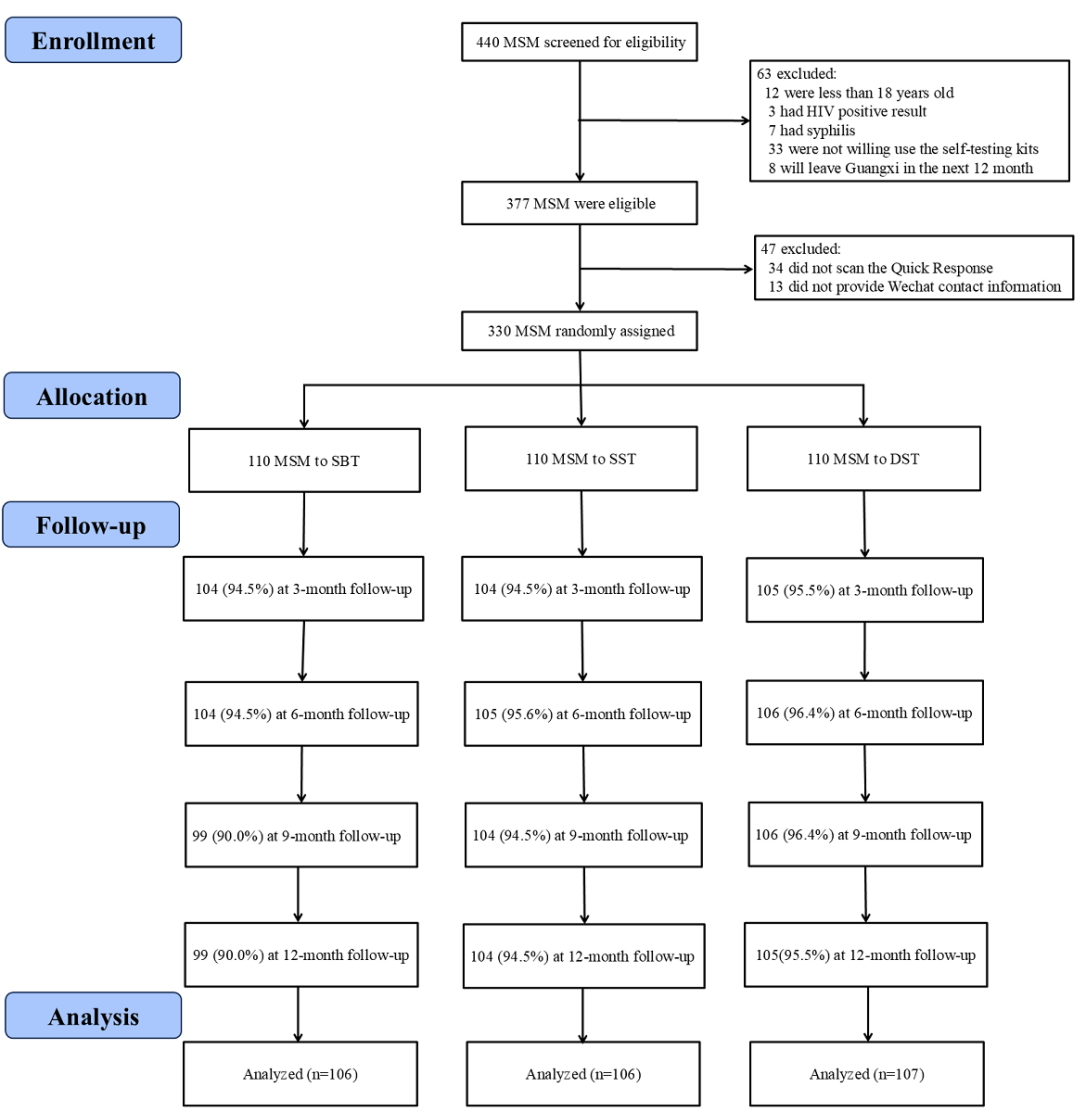
DST: dual HIV/syphilis self-testing group; MSM: men who have sex with men; SBT: site-based HIV testing group; SST: single HIV self-testing group.

Between September 1, 2021, and February 28, 2022, we assessed the eligibility of 440 MSM. Of these, 63 participants were excluded for not meeting the inclusion criteria, 34 did not scan the Quick Response Code, and 13 were excluded due to incomplete WeChat contact information. Ultimately, 330 eligible MSM were recruited and randomized into the SBT group (110/330, 33.3%), the SST group (110/330, 33.3%), and the DST group (110/330, 33.3%). After the 12-month study period, a total of 106 participants in the SBT group, 106 in the SST group, and 107 in the DST group completed at least 1 follow-up questionnaire and were included in the data analysis. The overall follow-up rate was 96.7% (319/330) (Figure 1).

### Baseline Sociodemographic and Behavioral Characteristics of MSM

A comprehensive overview of the baseline sociodemographic and behavioral characteristics is described in Table 1. All participants had similar baseline characteristics across the 3 arms. The majority of the cohort, comprising 73.4% (234/319) of participants, were aged between 18 and 30 years, and a substantial proportion (245/319, 76.8%) had a college education or above. Notably, 63.9% (204/319) had previous experience with HIVST. Furthermore, 43.9% (140/319) reported engaging in condomless anal and oral sex with male partners in the past 6 months.

**Table 1:** Baseline sociodemographic and behavioral characteristics of MSM^a^ enrolled in a pragmatic RCT^b^ of secondary HIV/syphilis self-testing distribution in Guangxi, China (between September 2021 and February 2022).

Characteristics		Overall (N=319), n (%)	SBT^c^ group (n=106), n (%)	SST^d^ group (n=106), n (%)	DST^e^ group (n=107), n (%)	*P* value	
**Age, year**							.254
	18-30	234 (73.4)	71 (67.0)	80 (75.5)	83 (77.6)		
	31-40	61 (19.1)	26 (24.5)	16 (15.1）	19 (17.8)		
	>40	24 (7.5)	9 (8.5)	10 (9.4）	5 (4. 7)		
**Ethnicity**							.912
	Han majority	207 (64.9)	69 (65.1)	67 (63.2)	71 (66.4)		
	Zhuang	96 (30.1)	32 (30.2)	32 (30.2)	32 (29.9)		
	Other	16 (5.0)	5 (4.7)	7 (6.6)	4 (3.7)		
**Insurance**							.797
	No	38 (11.9)	13 (12.3)	14 (13.2)	11 (10.3)		
	Yes	281 (88.1)	93 (87.7)	92 (86.8)	96 (89.7)		
**Highest level of education**						.064	
	High school or below	74 (23.2)	31 (29.2)	26 (24.5)	17 (15. 9)		
	College or beyond	245 (76.8)	75 (70.8)	80 (75.5)	90 (84.1)		
**Occupation**							.623
	Student	106 (33.2)	34 (32.1)	39 (36.8)	33 (30.8)		
	Nonstudent	213 (66.8)	72 (67.9)	67 (63.2)	74 (69.2)		
**Cohabitation**							.779
	Living with others	143 (44.8)	45 (42.5)	52 (49.1)	46 (43.0)		
	Living with family	79 (24.8)	29 (27.4)	25 (23.6)	25 (23.4)		
	Living alone	97 (30.4)	32 (30.2)	29 (27.4)	36 (33.6)		
**Monthly personal income**						.445	
	＜3000 RMB	135 (42.3)	50 (47.2)	44 (41.5)	41 (38.3)		
	3000-5000 RMB	83 (26.0)	29 (27.4)	24 (22.6)	30 (28.0)		
	＞5000 RMB	101 (31.7)	27 (25.5)	38 (35.8)	36 (33.6)		
**Marital status**							.934
	Unmarried	287 (90.0)	94 (88.7)	95 (89.6)	98 (91.6)		
	Married	23 (7.2)	8 (7.5)	8 (7.5)	7 (6.5)		
	Divorced or widowed	9 (2.8)	4 (3.8)	3 (2.8)	2 (1.9)		
**Ever used HIV self-testing**						.247	
	No	115 (36.1)	45 (42. 5)	35 (33.0)	35 (32.7)		
	Yes	204 (63.9)	61 (57.5)	71 (67.0)	72 (67.3)		
**Number of male sex partners in the past 6 months**							.389
	<2	134 (42.0)	44 (41.5)	51 (48.1)	39 (36.4)		
	2-5	157 (49.2)	55 (51.9)	45 (42. 5)	57 (53.3)		
	>5	28 (8.8)	7 (6.6)	10 (9.4)	11 (10.3)		
**Have male regular sex partners in the past 6 months**							.285
	No	158 (49.5)	48 (45.3)	59 (55.7)	51 (47.7)		
	Yes	161 (50.5)	58 (54.7)	47 (44.3)	56 (52.3)		
**Have male casual sex partners in the past 6 months**							.415
	No	132 (41.4)	43 (40.6)	49 (46.2)	40 (37.4)		
	Yes	187 (58.6)	63 (59.4)	57 (53.8)	67 (62.6)		
**Had a one-night stand with a male sex partner in the past 6 months**							.178
	No	139 (43.6)	46 (43.4)	53 (50.0)	40 (37.4)		
	Yes	180 (56.4)	60 (56.6)	53 (50.0)	67 (62.6)		
**Had condomless anal and oral sex with male partners in the past 6 months**							.625
	No	179 (56.1)	63 (59.4)	56 (52.8)	60 (56.1)		
	Yes	140 (43.9)	43 (40.6)	50 (47.2)	47 (43.9)		

^a^MSM: men who have sex with men.

^b^RCT: randomized controlled trial.

^c^SBT: site-based HIV testing.

^d^SST: single HIV self-testing.

^e^DST: dual HIV/syphilis self-testing.

### Primary Outcomes

Over 3 months, social network members in the intervention SST and DST groups were more likely to motivate others to undergo HIV testing compared with those in the SBT group. The mean number of motivated individuals was 0.42 in the SST group versus 0.20 in the SBT group (MD 0.22, 95% CI 0.12-0.33; *P*<.001), and 0.51 in the DST group versus 0.20 in the SBT group (MD 0.32, 95% CI 0.20–0.43; *P*<.001), both below the Bonferroni-corrected significance threshold of .017. However, there was no significant difference in the mean number of social network members tested per participant between the SST and DST groups over an average follow-up of 3 months (MD 0.09, 95% CI –0.05 to 0.23; *P*=.082), which is above the Bonferroni-corrected significance threshold of .017 (Table 2).

**Table 2:** Secondary distribution outcomes: mean number of social network members motivated to test for HIV by study arm in a pragmatic RCT^a^ of secondary HIV/syphilis self-testing distribution among MSM^b^ in Guangxi, China (3-month average duration between 2021 and 2023).

Outcomes	SBT^c^ group	SST^d^ group	DST^e^ group	SST versus SBT			DST versus SBT			DST versus SST	
	Mean (95% CI)	Mean (95% CI)	Mean (95% CI)	MD^f^ (95% CI)	*P* value^g^	MD (95% CI)		*P* value	MD (95% CI)		*P* value
Total number of social network members	0.20 (0.15-0.26)	0.42 (0.34-0.52)	0.51 (0.42-0.63)	0.22 (0.12-0.33)	<.001	0.32 (0.20-0.43)		<.001	0.09 (–0.05 to 0.23)		.187
Via self-testing kits	0.09 (0.06-0.13)	0.27 (0.20-0.36)	0.35 (0.27-0.46)	0.18 (0.10-0.27)	<.001	0.27 (0.16-0.37)		<.001	0.08 (–0.04 to 0.21)		.175
Via facility-based testing	0.11 (0.07-0.15)	0.14 (0.10-0.18)	0.16 (0.11-0.20)	0.03 (–0.03 to 0.08)	.300	0.05 (–0.01 to 0.10)		.100	0.02 (–0.04 to 0.08)		.541

^a^RCT: randomized controlled trial.

^b^MSM: men who have sex with men.

^c^SBT: site-based HIV testing.

^d^SST: single HIV self-testing.

^e^DST: dual HIV/syphilis self-testing.

^f^MD: mean difference.

^g^*P* values are interpreted against a Bonferroni-corrected threshold of .017 for the 3 pairwise comparisons per outcome.

Using the Bonferroni-corrected threshold of .017, the mean frequency of total HIV tests per participant in the SST group (1.33) was higher than that in the SBT group (0.87; MD=0.46, 95% CI 0.31-0.62; *P*<.001). This disparity was mainly due to the difference in HIVST between the 2 groups (SST 0.88 vs SBT 0.33; MD=0.56, 95% CI 0.41-0.70; *P*<.001). Conversely, the mean frequency of facility-based HIV testing was comparable between SST (0.43) and SBT (0.51) (MD=–0.09, 95% CI –0.18 to 0.01; *P*=.07). Similarly, the mean frequency of total HIV tests per participant in the DST group (1.43) surpassed the mean (0.87) in the SBT group (MD=0.57, 95% CI 0.41-0.73; *P<*.001) within a 3-month average duration. This difference was predominantly attributed to the difference in HIVST between the 2 groups (DST 0.94 vs SBT 0.33; MD=0.61, 95% CI 0.46-0.75; *P*<.001). Notably, the mean frequency of facility-based HIV testing was also comparable between the DST (0.47) and SBT (0.51) group (MD=–0.04; 95% CI –0.14 to 0.06; *P*=.398). However, there was no difference between the SST and DST groups in the mean frequency of either total, self-tests, or site-based tests (all *P*>.017) (Table 3).

**Table 3:** Frequency of HIV testing per participant by study arm in an RCT^a^ of secondary HIV/syphilis self-testing distribution among MSM^b^ in Guangxi, China (3-month average duration between 2021 and 2023).

Outcomes	SBT^c^ group	SST^d^ group	DST^e^ group	SST versus SBT			DST versus SBT			DST versus SST	
	Mean (95% CI)	Mean (95% CI)	Mean (95% CI)	MD^f^ (95% CI)	*P* value^g^	MD (95% CI)		*P* value	MD (95% CI)		*P* value
Total Frequency of HIV tests	0.87 (0.77-0.97)	1.33 (1.21-1.45)	1.43 (1.31-1.56)	0.46 (0.31-0.62)	<.001	0.57 (0.41-0.73)		<.001	0.10 (–0.07 to 0.28)		.245
Via self-testing kits	0.33 (0.27-0.40)	0.88 (0.77-1.01)	0.94 (0.82-1.07)	0.56 (0.41-0.70)	<.001	0.61 (0.46-0.75)		<.001	0.05 (–0.13 to 0.23)		.555
Via facility-based testing	0.51 (0.44-0.58)	0.43 (0.36-0.49)	0.47 (0.41-0.54)	–0.09 (–0.18 to 0.01)	.070	–0.04 (–0.14 to 0.06)		.398	0.05 (–0.05 to 0.14)		.328

^a^RCT: randomized controlled trial.

^b^MSM: men who have sex with men.

^c^SBT: site-based HIV testing.

^d^SST: single HIV self-testing.

^e^DST: dual HIV/syphilis self-testing.

^f^MD: mean difference.

^g^*P* values are interpreted against a Bonferroni-corrected threshold of .017 for the 3 pairwise comparisons per outcome.

### Secondary Outcomes

Over the 12-month follow-up, 26 individuals were diagnosed with HIV, including 4 (15.4%) participants and 22 (84.6%) social network members. Of the 22 social network members diagnosed with HIV, 1 (4.5%) was from the SBT group, 12 (54.5%) were from the SST group, and 9 (40.9%) were from the DST group. Regarding syphilis diagnoses, 11 participants were positive, with 1 (9.1%) from the SBT group, 3 (27.3%) from the SST group, and 7 (63.6%) from the DST group. All individuals identified as living with HIV or syphilis positive underwent laboratory-based confirmation tests and were linked to treatment through the national HIV or syphilis control program. There were no statistically significant differences between participants in the 3 study groups regarding HIV and syphilis positive test outcomes and social network members with an HIV positive test (Table 4).

**Table 4:** Identified HIV and syphilis infection among participants and their social network members during the 12-month follow-up period in a pragmatic RCT^a^ of secondary HIV/syphilis self-testing distribution among MSM^b^ in Guangxi, China (2021-2023).

Outcomes	SBT^c^	SST^d^	DST^e^	SST versus SBT		DST versus SBT			DST versus SST	
	n/N (%; 95% CI)	n/N (%; 95% CI)	n/N (%; 95% CI)	RD^f^ (95% CI)	*P* value^g^	RD (95% CI)	*P* value	RD (95% CI)		*P* value
HIV positive	1/106 (0.94; –0.93 to 2.81)	1/106 –0.93 to 2.81)	2/107 (1.87; –0.74 to 4.48)	—^h^	—	0.93 (–3.16 to 5.01)	.620	0.93 (–3.16 to 5.01)		.620
Syphilis positive	1/106 (0.94; –0.93 to 2.81)	3/106 (2.83; –0.38 to 6.04)	7/107 (6.54; 1.78-11.30)	1.89 (–2.71 to 6.48)	0.624	5.60 (–0.37 to 11.57)	.038	3.71 (–2.88 to 10.30)		.202
HIV positive results of social network members	1/126 (0.79; –0.77 to 2.36)	12/245 (4.90; 2.18-7.62)	9/309 (2.90; 1.03-4.80)	4.10 (0.39-7.82)	0.019	2.12 (–0.87 to 5.11)	.460	–1.99 (–5.64 to 1.67)		.341

^a^RCT: randomized controlled trial.

^b^MSM: men who have sex with men.

^c^SBT: site-based HIV testing.

^d^SST: single HIV self-testing.

^e^DST: dual HIV/syphilis self-testing.

^f^RD: risk (probability) difference (expressed as a percentage).

^g^*P* values are interpreted against a Bonferroni-corrected threshold of .017 for the 3 pairwise comparisons per outcome.

^h^Data not available.

## Discussion

### Principal Findings

Our pragmatic RCT evaluated whether secondary distribution strategy of dual HIV/syphilis self-testing and HIVST could increase HIV testing coverage by facilitating kit distribution within social networks, thereby addressing a critical evidence gap in LMICs. This approach contributes significantly to achieving the initial “95%” target outlined by UNAIDS [27]. While the absolute increase in motivated social network members per participant was modest at the individual level, prior cost-effectiveness analyses demonstrate that such incremental gains translate to meaningful population-level impacts. Wang et al [22] showed that dual HIV/syphilis self-testing among Chinese MSM is cost-efficient, with a cost per person tested substantially lower for dual HIV/syphilis self-testing (US $26.55) compared to standard care (US $66.19), and an incremental cost of US $17.55 per additional person tested. This confirms the efficiency of scaling such interventions in LMICs.

Notably, previous secondary distribution programs of HIVST in China have demonstrated the effect of this strategy in expanding testing coverage [28]. Similarly, a study conducted in California suggests that a network-based strategy for HIVST distribution is a promising intervention to increase testing uptake and reduce undiagnosed infections among MSM [29]. Moreover, monetary incentives, either independently or in conjunction with peer referrals, have proven effective in promoting the secondary distribution of HIVST among MSM [28]. A previous study in China also suggests that integrating social media with the secondary distribution of self-test kits holds promise to increase HIV/syphilis testing coverage and case identification among MSM [21]. These findings carry significant implications for the expansion of HIVST among key populations and may be applicable to other areas of public health research.

Our results also indicate that dual HIV/syphilis self-testing and HIVST can increase the frequency of total HIV testing in MSM, consistent with findings from other studies [26,30,31]. In contrast to the SBT group, both the SST and DST groups increased the frequency of total HIV testing among MSM in China. As the differences in the average frequency of site-based HIV testing among the 3 groups were similar, the higher average frequency of total HIV testing in both the SST and DST groups can be mainly attributed to their increased average frequency of HIVST compared to the SBT group. This shift did not reduce facility-based testing, indicating self-testing complements rather than replaces clinical services.

Our findings indicate that dual HIV/syphilis self-testing can yield results similar to single HIVST in the average frequency. The integration of HIV and syphilis self-testing has the potential to strengthen public health interventions aimed at addressing HIV and syphilis control among MSM [14]. Despite syphilis being a significant public health challenge, it is often overlooked and underfunded, particularly in LMICs [32,33]. Previous research indicates that only 30% of MSM in China have ever undergone a syphilis test [9]. The anticipated stigma associated with syphilis may impede testing efforts [34]. Therefore, syphilis self-testing emerges as a potentially effective approach to overcoming these barriers [9,35]. Crucially, DST achieved comparable HIV testing outcomes to SST while simultaneously enabling syphilis screening. Given the low HIV and syphilis testing uptake in LMICs, it is crucial to use dual HIV/syphilis self-testing to increase testing coverage.

A systematic review has demonstrated the acceptability and cost-effectiveness of integrating syphilis self-testing into HIVST services [14]. It also significantly increases syphilis testing among MSM in China, demonstrating cost-effectiveness [22]. Building upon this evidence base, our trial uniquely focuses on the secondary distribution of dual HIV/syphilis self-testing among MSM. To our knowledge, this is the first RCT to directly compare DST with SST within a network-based distribution model. Our findings demonstrate that DST increased HIV testing frequency comparably to SST and led to the identification of more undiagnosed syphilis cases (7 in DST vs 3 in SST), highlighting its potential to address the syndemic transmission of sexually transmitted infections (STIs) in LMICs where syphilis testing is neglected. This highlights the significant added value of DST beyond HIVST alone. However, the DST group's numerical increase in HIV or syphilis positivity among participants and their network members did not reach statistical significance compared to other groups. This lack of statistical significance is likely attributable to insufficient statistical power due to the relatively small number of incident cases and the observation period of this trial. Therefore, further research with larger sample sizes and longer follow-up is needed to robustly assess the impact of DST distribution on HIV and syphilis incidence.

Despite demonstrated efficacy in secondary DST distribution, scaling this intervention across LMICs necessitates addressing practical barriers—particularly cost constraints for sustained kit provision and infrastructure limitations in remote regions. Mitigation strategies include integrating with existing public health systems such as CBOs and CDC clinics to leverage their established networks for efficient distribution, confirmatory testing, and care linkage, thereby reducing operational costs. Further cost efficiency may be achieved through the bulk procurement of dual self-test kits instead of separate HIV/syphilis tests and by establishing public-private partnerships for subsidized pricing. Implementation should prioritize phased geographical scale-up, beginning with urban hubs possessing CBO partnerships before extending to periurban or rural areas.

### Limitations

Our study has several limitations. First, this RCT recruited participants mainly through CBO referrals and social media, focusing on individuals who volunteered for HIV testing at CBOs and expressed a willingness to use HIVST. This recruitment approach may have excluded MSM who were averse to attending site-based testing at CBOs or hesitant to adopt HIVST, particularly those with lower educational attainment or limited digital access. Consequently, our findings are primarily generalizable to MSM engaged with CBOs or actively seeking testing services and may not fully represent the broader MSM population in China, including hidden high-risk subgroups with socioeconomic vulnerabilities. Future studies should employ diversified recruitment strategies (eg, venue-based sampling or peer-driven recruitment in rural areas) to include MSM with diverse educational backgrounds. Additionally, hybrid interventions combining online platforms and offline community support may enhance accessibility for populations with limited literacy or digital resources. Second, the implementation of this RCT coincided with the COVID-19 lockdown period in China, particularly during the 6-month follow-up, disrupting HIV services. The impact of this disruption may have enhanced the uptake of self-testing. Third, social desirability and self-report biases may influence the accuracy of self-reported testing frequencies and sexual behaviors. Although we implemented mitigation strategies, including anonymized data collection and photo verification of self-test results, residual bias could persist. The study findings could be susceptible to social desirability reporting bias, as participants were required to self-report sensitive personal information. Fourth, the absence of a limit on the number of testing kits participants could obtain might lead to unnecessary testing during the experiment. Our original intention, allowing individuals to apply for multiple kits, aimed to encourage participants to distribute more kits within their social network. Fifth, although we observed sustained effects over 12 months, the long-term durability beyond 1 year remains uncertain. Future studies with longer follow-up are needed to assess whether the effects persist without the ongoing provision of free self-testing kits. Sixth, the lack of detailed syphilis outcomes—such as treatment initiation, serological outcomes after diagnosis, or longitudinal follow-up—restricts our ability to fully evaluate the comparative effectiveness of DST versus SST in improving syphilis control. Future studies should prioritize comprehensive syphilis outcome metrics to validate the public health utility of dual testing.

### Conclusions

The secondary distribution strategy of HIV and dual HIV/syphilis self-testing effectively expanded HIV testing coverage among MSM in China by facilitating kit distribution within social networks. Both strategies increase HIV testing frequency. These findings provide policymakers with evidence to support the incorporation of self-testing into national HIV and STI control programs, particularly in resource-limited settings, as a cost-effective approach to improve testing coverage. Future studies should explore cost-effective implementation methods in high-risk MSM.

## Appendix

Multimedia Appendix 1 CONSORT (Consolidated Standards of Reporting Trials) 2010 checklist.

## Abbreviations

CBO: community-based organization
CDC: Centers for Disease Control and Prevention
DST: dual HIV/syphilis self-testing
HIVST: HIV self-testing
LMIC: low- and middle-income country
MD: mean difference
MSM: men who have sex with men
RCT: randomized controlled trial
RD: risk difference
SBT: site-based HIV testing
SST: single HIV self-testing
STI: sexually transmitted infection
UNAIDS: United Nations Programme on HIV/AIDS
WHO: World Health Organization

## Data Availability

The deidentified data sets generated during the study, along with the statistical analysis plan and analytic code, can be made available from the corresponding author on reasonable request.
